# High level SARS-CoV-2 nucleocapsid refolding using mild condition for inclusion bodies solubilization: Application of high pressure at pH 9.0

**DOI:** 10.1371/journal.pone.0262591

**Published:** 2022-02-03

**Authors:** Rosa Maria Chura-Chambi, Alvaro Rossan de Brandão Prieto-da-Silva, Matheus Martins Di Lela, João Ezequiel Oliveira, Patricia Estima Antonia Abreu, Luciana Regina Meireles, Heitor Franco de Andrade Junior, Ligia Morganti

**Affiliations:** 1 Centro de Biotecnologia, Instituto de Pesquisas Energéticas e Nucleares, IPEN-CNEN/SP, São Paulo, SP, Brazil; 2 Laboratório de Genética, Instituto Butantan, São Paulo, SP, Brazil; 3 Laboratório de Bacteriologia, Instituto Butantan, São Paulo, SP, Brazil; 4 Laboratório de Protozoologia, Instituto de Medicina Tropical de São Paulo, Universidade de São Paulo, São Paulo,SP, Brazil; East China Normal University School of Life Sciences, CHINA

## Abstract

SARS-CoV-2 Nucleocapsid (N) is the most abundant viral protein expressed in host samples and is an important antigen for diagnosis. N is a 45 kDa protein that does not present disulfide bonds. Intending to avoid non-specific binding of SARS-CoV-2 N to antibodies from patients who previously had different coronaviruses, a 35 kDa fragment of N was expressed without a conserved motif in *E*. *coli* as inclusion bodies (N122-419-IB). Culture media and IB washing conditions were chosen to obtain N122-419-IB with high yield (370 mg/L bacterial culture) and protein purity (90%). High pressure solubilizes protein aggregates by weakening hydrophobic and ionic interactions and alkaline pH promotes solubilization by electrostatic repulsion. The association of pH 9.0 and 2.4 kbar promoted efficient solubilization of N122-419-IB without loss of native-like tertiary structure that N presents in IB. N122-419 was refolded with a yield of 85% (326 mg/L culture) and 95% purity. The refolding process takes only 2 hours and the protein is ready for use after pH adjustment, avoiding the necessity of dialysis or purification. Antibody binding of COVID-19-positive patients sera to N122-419 was confirmed by Western blotting. ELISA using N122-419 is effective in distinguishing between sera presenting antibodies against SARS-CoV-2 from those who do not. To the best of our knowledge, the proposed condition for IB solubilization is one of the mildest described. It is possible that the refolding process can be extended to a wide range of proteins with high yields and purity, even those that are sensible to very alkaline pH.

## Introduction

Coronavirus disease 2019 (COVID-19) is caused by the highly contagious severe acute respiratory syndrome coronavirus 2 (SARS-CoV-2). The SARS-CoV-2 is composed of a large single-stranded positive-sense RNA genome of approximately 30,000 nucleotides, which contains two flanking untranslated regions and 13–16 open reading frames (ORFs) encoding non-structural and structural proteins including spike (S), envelope (E), membrane (M), nucleocapsid (N) and accessory proteins [1]. Genomic analysis of the new SARS-Cov-2 virus showed a high similarity with SARS-CoV (about 79%), lower similarity with MERS-CoV (about 50%) and still lower similarity to common flu coronaviruses [2].

The primary functions of SARS-CoV-2 N protein are binding to the viral RNA genome and packing them into a long helical nucleocapsid structure or ribonucleoprotein (RNP) complex. N protein binds to leader RNA, and maintains highly ordered RNA conformation, suitable for replicating and transcribing the viral genome [3]. This protein consists of three distinct domains: a N-terminal domain, NTD (46–174), that is responsible for RNA binding, an intrinsically disordered central Ser/Arg (SR)-rich linker (183–210) for primary phosphorylation and a C-terminal dimerization domain, CTD (258–361) responsible for oligomerization that is also a RNA-binding domain. N is a highly basic protein containing an excess of positive charges. These charges are considered important for RNA binding, but they are also potentially deterring for the self-association of the protein through electrostatic repulsion [4,5].

The N protein is the most abundant viral protein expressed in host samples during the early stages of infection [6]. Furthermore, proteins S and N are efficient epitopes to activate B and T lymphocytes immune response [7]. Therefore, these regions are suitable targets for immune recognition of SARS-CoV-2 in immunodiagnostic and as vaccine antigens. N protein presents three B cell immunodominant regions (43–65, 154–175 and 365–403 residues) [8,9], including a highly conserved motif (FYYLGTGP) occurring in the N-terminal [10].

Diagnosis of COVID-19 is a critical step in tracing the virus and understanding its epidemiology. Molecular biology tests used to detect specific regions of the viral genome, such as Reverse-Transcription Polymerase Chain Reaction (RT-PCR), is a gold standard method for the diagnosis of SARS-CoV-2, but they are dependent on the presence of the viral RNA to give positive result. Immunological assays such as Enzyme Linked Immunosorbent Assay (ELISA) determine specific IgM and/or IgG antibody against SARS-CoV-2 nucleocapsid N, S1 or receptor binding domain (RBD) of S. The ELISA for the detection of antibodies against N and RBD were described to be more sensitive but less specific than S1 antibodies. Reports in the literature suggest that false-positive ELISA results may occur with SARS-CoV-1 infection as the protein N is extremely conserved among human-infecting β-coronavirus [11].

A SARS-CoV recombinant N protein lacking the first 119 amino acid residues, which excluded the conserved domain in order to prevent cross-binding of antibodies against other coronaviruses, was used by Tan et al in an ELISA assay. All sera from SARS-CoV convalescent patients showed immunoreactivity towards N120-422 while the serum from none of the healthy donors presented reaction [12].

Recombinant proteins expressed in *Escherichia coli* often form aggregates in the cytoplasm, as inclusion bodies (IB). Secondary and tertiary structures similar to those found in the native conformation are often found in IB [13]. Frequently used refolding methods employing high concentrations of denaturing agents for solubilization of proteins in IB causes the loss of native-like structures and re-aggregation due to intermolecular interactions among exposed hydrophobic domains [14]. The use of high pressure (2–5 kbar) can enhance the solubilization of aggregates by disrupting ionic and hydrophobic interactions without denaturation [15], but efficient solubilization is usually obtained only by simultaneously modifying other conditions, such as the addition of low concentration of a chaotropic reagent [16]. Alkaline pH promotes the breakdown of intermolecular bonds by electrostatic repulsion [17] but efficient solubilization is often obtained at extremely basic pH or in the presence of another solubilization reagent [18]. We have previously demonstrated an efficient and non-denaturing IB solubilization of dengue and zika NS1 proteins, through the combination of high pressure and alkaline pH, which dramatically diminished re-aggregation [19,20]. This mild IB solubilization process usefully maintains native-like structures that are generally present in IB which is suitable for subsequent refolding.

In the present study, we produced high level expression of the fragment 122–419 of SARS-CoV-2 N protein as inclusion bodies (IB) in *Escherichia coli*. The resulting protein is 35 kDa and does not contain disulfide bonds. The IBs were solubilized and refolded under mild condition of pH 9.0 and high pressure. The protein was shown to be suitable as antigen in an ELISA assay to detect IgG antibodies against SARS-CoV-2 in COVID-19 patients.

## Materials and methods

### N122-419 cloning

The DNA containing optimized codons for the fragment 122–419 of the SARS-CoV-2 gene (Genebank MT126808.1, protein id. QIG56001.1), flanked by two 6His tags at N- and C-terminal was synthetized and cloned in pET-28 (Merck) by Genscript. Sequence analysis and multiple sequence alignment of viral nucleoproteins were performed using Omega-Clustal and Jalview workbench [21].

### Expression and isolation of N122-419-IB

BL21(DE3) bacteria transformed with the plasmid pET28-N122-419 were plated in LB agar containing kanamycin. Selected bacterial clones were inoculated in 1 L of the rich medium 2-fold HKSII: yeast extract, 10 g/L; tryptone, 20 g/L; acid hydrolyzed casein, 4 g/L; K_2_SO_4_, 3.1 g/L, MgSO_4_, 0.8 g/L, CaCl_2_, 0.08 g/L and 0.32 ml/L solution of trace metals (Fe, Zn, Mn, Cu, Co, B, Mo, and I) [22] and incubated at 37° C in a rotary shaker. When optical density at 600 nm reached 2.5–3.0, IPTG was added to the final concentration of 0.5 mM. The temperature was decreased to 30° C and agitation continued for a further 16 h. For the isolation of the IB, the culture was centrifuged at 4°C at 3,000 x g for 15 min and the supernatant was discarded. The pellet was resuspended in 50 mL 0.1 M Tris HCl pH 7.0, 5 mM EDTA and 1 mM PMSF. Lysozyme was added to a final concentration of 50 μg/mL and the suspension was incubated for 15 minutes at room temperature. Sodium deoxycholate was added to 0.1% and bacteria were lysed by intermittent sonication with the suspension kept in ice to avoid over-heating the material, until it completely lost viscosity. The suspension was centrifuged at 15,000 x g for 15 minutes at 4°C; supernatant was discarded and the pellet was suspended in 50 mL of 0.1 M Tris HCl pH 7.0 containing 0.1% sodium deoxycholate, 1 mM PMSF and 5 mM EDTA and sonicated to disrupt the lumps. The suspension was centrifuged and washed again. The pellet was suspended in 50 mL 50 mM Tris HCl buffer at pH 7.0 containing 1 mM EDTA and 1 mM PMSF, centrifuged and suspended in 20 mL of the same buffer. The absorbance at 280 nm of the N122-419-IB solubilized in 6M GdnHCl was used for determination of protein concentration assuming that 3.3 OD correspond to 1 mg/ml. Aliquots of the suspension were kept in a -20°C freezer. When the aliquots were thawed, they were sonicated again to disrupt the lumps.

### Determination of N122-419 protein concentration

Bradford protein assay and SDS-PAGE using BSA as standard were performed, as well as determination of Absorbance at 280 nm. We found that a concentration of 1 mg/ml of N122-419 verified by Bradford assay and SDS-PAGE corresponds to A_280 nm_ of 3.3. Therefore absorbance at 280 nm was used for measurement of N122-419 concentration in the tests performed.

### Solubilization of N122-419-IB at high hydrostatic pressure and at 1 bar

For solubilization we used 1 mL of N122-419-IB suspension diluted to a concentration of 1.1 mg N122-419/mL in buffers 50 mM Tris HCl for pH 7.0 to 9.0 and 50 mM CAPS for pH 10.0 to 11.0 with 1 mM EDTA. The suspensions were introduced into plastic bags that were sealed and placed into a larger plastic bag that was vacuum-sealed. The bag was put into a pressure vessel (R4-6-40, High Pressure Equipment, USA) and pressurized to 2.4 kbar for 90 min using oil as a transmission fluid in a suitable high-pressure pump (PS-50, High Pressure Equipment, USA) at 20°C. The pH of the supernatant was then lowered by dilution to 0.22 mg/mL in Tris HCl 50 mM pH 7.5.

### Fluorescence and light scattering (LS)

Analysis of N122-419 tertiary structure by exposure of Trp residues was performed by intrinsic fluorescence. Analysis of N122-419 solubilization was determined by LS, because the insoluble IB promotes scattering of the light and a drop in LS values therefore indicates the solubilization of the aggregates. Cary Eclipse spectrofluorimeter (Varian) was used for Fluorescence and LS determinations. Measurements were performed at a 90° angle relative to the incident light using a 1 second response time and reading speed of 240 nm/minute. Data were collected using 1 cm optical path cuvettes. Excitation at 280 nm was utilized for intrinsic fluorescence determination between 300 and 400 nm. LS measurements were performed with excitation at 320 nm, and light scattering was collected from 315 to 325 nm. The program Origin 9.1 was used to construct the fluorescence graphics and the value of the peak of maximal fluorescence (λ_max)_ was determined using smoothed curves.

### Serum samples

This study was performed using human serum samples obtained from the biorepository collection of the Laboratory of Protozoology of the Institute of Tropical Medicine, University of São Paulo (IMT/USP), São Paulo, Brazil. Positive samples (n = 61) were obtained from volunteers who were diagnosed with COVID-19 using molecular testing (RT-qPCR) and/or based on the presence of anti-SARS-CoV-2 IgGs. Serum samples placed in the biorepository before COVID-19 pandemic were used as negative control (n = 50). All procedures were approved by the Research Ethics Committee of Hospital das Clínicas, School of Medicine of the University of São Paulo (HCFMUSP) (São Paulo, Brazil)(protocol number: 31685020.4.0000.0068). All research participants were older than 18 year old and they provided written informed consent prior to inclusion in this study.

### Immunoblotting

N122-419 protein (1.1μg/lane) was subjected to SDS-PAGE (12% Mini-PROTEAN TGX Precast Protein Gel, Bio-Rad) under reducing conditions and transferred to a nitrocellulose membrane. Nonspecific binding sites were blocked by using 10% (w/v) dry milk in PBS-0.05% Tween (PBS-T) at 4°C. The membrane was incubated with human serum samples (diluted 1:500) or with mouse antibody (diluted 1:10,000) as a positive control to recognize N122-419 protein. After three washes with PBS-T, the membrane was incubated with a secondary peroxidase-conjugated anti-human IgG or anti-mouse antibodies at a 1:5,000 dilution. The membrane was washed three times with PBS-T after the incubation. Positive signals were detected using enhanced chemiluminescence Western blot detection reagents (Thermo Fisher Scientific, Boston, MA, USA).

### Analysis of the bands in SDS-PAGE

Analysis of bands in SDS-PAGE for the determination of N122-419 purity and for the refolding yield calculation was performed using Image J program.

### Enzyme linked immunosorbent assay (ELISA)

The detection of anti-SARS-CoV-2 IgG antibodies was performed using an in-house ELISA as described elsewhere by Meireles et al. [23]. Briefly, a 96 well microplate (Corning^®^, New York, USA) was coated with 100 μl of N122-419 protein (2.2 μg/mL) diluted in 0.1 M sodium carbonate buffer pH 9.5 and the microplates were maintained overnight at 4°C in a humid chamber. The microplates were washed with PBS containing 0.05% Tween 20 (PBST) and blocked with 250 μL of 5% fat-free milk (Molico^®^) diluted in PBST for 1h at 37°C. After washing, 100 μL of serum samples diluted 1:100 in PBST containing 5% fat-free milk (PBST-milk) were incubated with viral antigens for 1h at 37°C. The microplates were again washed and 100 μL/well of anti- human IgG peroxidase conjugate (Sigma, St. Louis, USA) diluted 1:20,000 in PBST-milk were added and incubated for 1h at 37°C. After washing, 100 μL/well of ABTS substrate solution (2, 2′-Azino-bis (3-ethylbenzothiazoline-6-sulfonic acid) were added. The microplates were maintained in the dark for 30 min at room temperature. The reaction was stopped with 0.1M citric acid (100 μL/well). Optical density (OD) at 414 nm was measured in a microplate reader (Labsystems Multiskan MS^®^, Midland, Canada). ELISA results were expressed in OD at 414 nm and the cut-off threshold was determined using the receiver operating characteristic (ROC) curve.

## Results

### Homology of N

**Fig 1:**
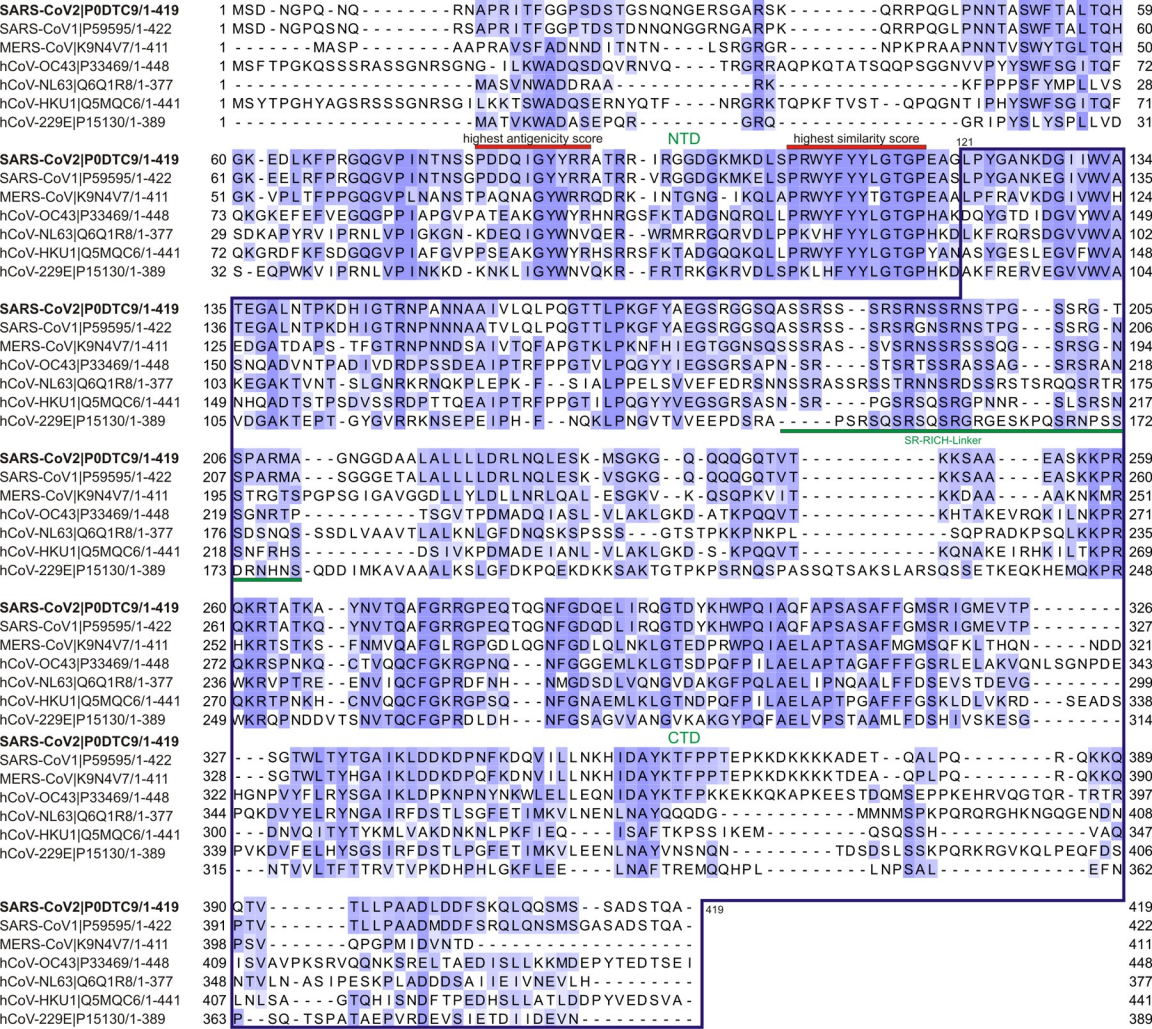
Amino acid sequence conservation of predicted linear peptide epitope sequences from human coronavirus nucleocapsid proteins (N). JalView Clustal Omega multiple sequence alignment of viral nucleoproteins are shown across all five known human β-coronaviruses: SARS-CoV-2 (P0DTC9); SARS-CoV (P59595); hCoV-HKU1 (Q5MQC6); hCoV-OC43 (P33469); MERS-CoV (K9N4V7) and two known human α-coronaviruses: hCoV-229E (P15130) and hCoV-NL63 (Q6Q1R8). Each row in the depicted alignments corresponds to the protein sequence from the indicated coronavirus identified by UniprotKB accession numbers with the starting position of the viral amino acid sequence shown at left and position coordinates of the overall blue shading indicates the extent of sequence similarity by BLOSUM62 score, with the darkest blue shading indicating a 100% match for that amino acid across all sequences. The red-highlighted sequences correspond to highly conserved and highest antigenicity predicted epitope peptides scores. NTD: N-terminal domain or RNA binding domain and the CTD: C-terminal domain or dimerization domain are separated by a SR-Rich-Linker delimitated by a green bar. The blue outline box delineates the corresponding coding sequence cloned to express the fragment of nucleocapsid protein used to detect specific antibodies against SARS-CoV-2 N122-419.

Fig 1 presents the alignment of SARS-CoV-2 with SARS-CoV and other types of inconsequential coronaviruses that are responsible for the common cold. The domain FYYLGTGP, which is highly conserved among these viruses, is located at residues 110–117 in SARS-CoV-2 [24]. Therefore, we decided not to include the codons of the first 121 residues in the synthesized gene to avoid non-specific binding of antibodies produced against other coronaviruses to the common domain.

### Solubilization and refolding of N122-419-IB

**Fig 2:**
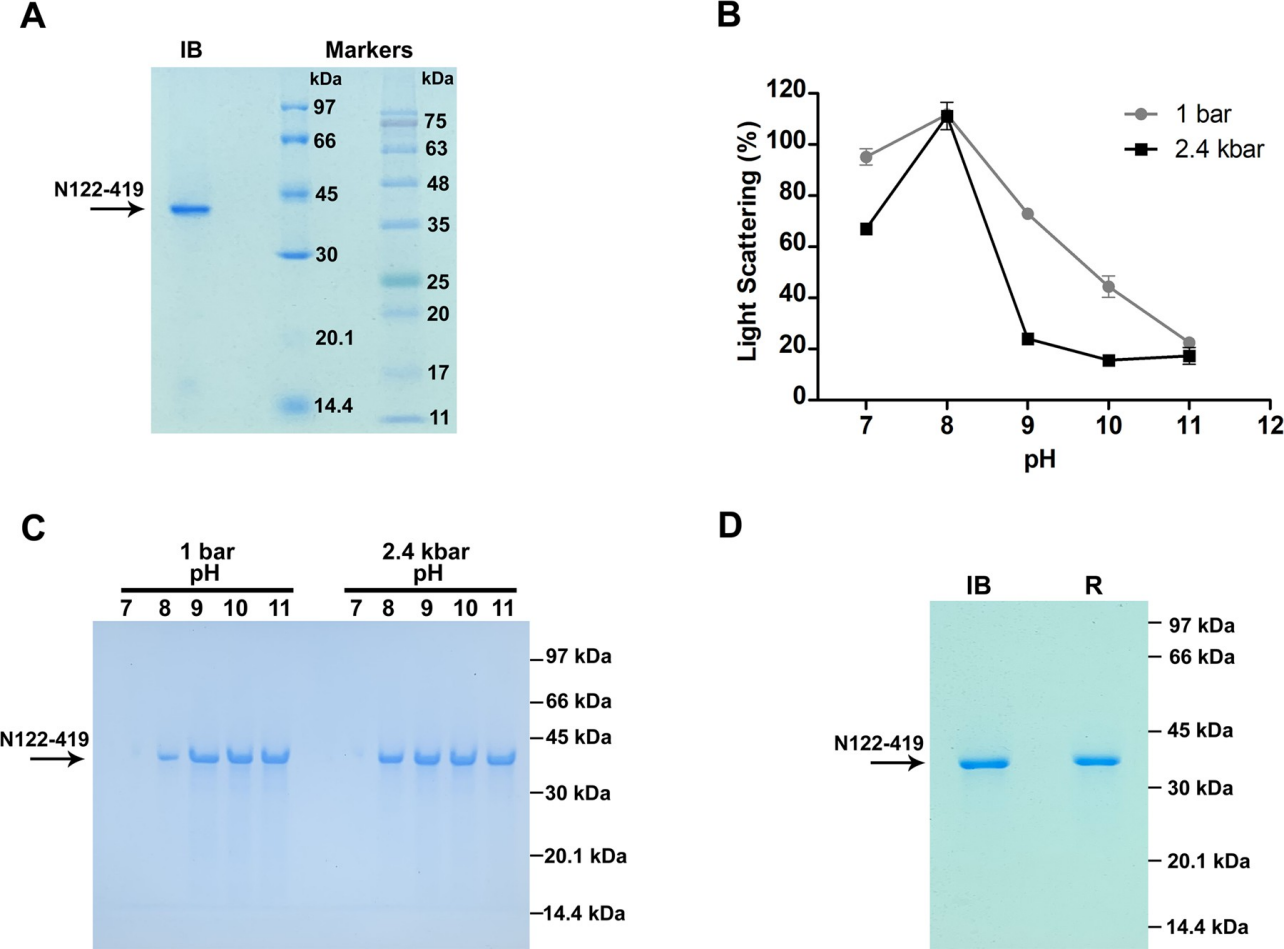
Compression at 2.4 kbar and alkaline pH promote solubilization of N122-419-IB. Suspensions of N122-419-IB were subjected to 2.4 kbar for 90 min or to 1 bar for 4 h. A, SDS-PAGE of N122-419-IB; B, Light scattering (LS) vs pH. Values are expressed as LS mean ± SD. The LS value, obtained for the suspension at a pH 7.0 at 1 bar was considered to be 100%. Each condition was analyzed in duplicate; C, SDS-PAGE of fractions of N122-419-IB that were solubilized at different pH; D, SDS-PAGE of N122-419-IB (IB) and refolded N122-419 (R). All samples were reduced. The results are representative of 2 independent assays.

A rich medium (2-fold HKSII) containing high concentration of amino acids was utilized to allow production of high level of N122-419 by transformed *Escherichia coli*. N122-419-IBs were extracted from the disrupted bacteria and washed with sodium deoxycholate detergent to reduce bacterial contaminants. We obtained a suspension of N122-419-IB that contained a high level of N, corresponding to 369 mg/L bacterial culture presenting a high purity, of more than 90%, as determined by analysis of the N122-419-IB band in SDS-PAGE (Fig 2A).

The association of high pressure and alkaline pH was previously shown to be effective to solubilize aggregated proteins within IBs [20]. In the present study we determined the minimal pH able to solubilize N122-419-IB at high pressure and at 1 bar. Increased solubilization were obtained in suspensions subjected to 2.4 kbar for 90 min, as indicated by lower values of visible light scattering (LS), than for samples incubated at 1 bar for 4 h, the time required for LS stabilization (Fig 2B). The exception was at pH 8.0 that showed a higher value of LS than at pH 7.0, possibly explained by formation of smaller aggregates that are more effective to scatter the light. As expected, N122-419 becomes more soluble as the pH increases and is obtained in the supernatants of the IB suspensions starting from pH 8.0 and higher, as also confirmed by the presence of N122-419 35 kDa bands in SDS-PAGE (Fig 2C). The protein was efficiently solubilized at pH 9 and 2.4 kbar, which was chosen as a solubilization condition. Refolding was performed simply by diluting the solubilized N122-419 to a lower pH. Efficient N122-419-IB solubilization can be obtained at a protein concentration of up to 4.3 mg/mL. Dialysis was not necessary because solubilization was obtained in the absence of additives. The results indicate that, despite the fact that solubilization was improved by the effect of high pressure, solubilization can be performed at atmospheric pressure, at a higher pH, 10.0 or 11.0. Significant reaggregation to insoluble clusters was not observed and the refolding yield was of approximately 85% in relation to the amount of protein within N122-419-IB, as determined by SDS-PAGE (Fig 2C) and by absorbance measurements at 280 nm. The yield of 326 mg refolded N122-419 was obtained from 1 L bacterial culture, with a purity of approximately 95% (Fig 2D), as determined by analysis of bands in SDS-PAGE.

### Effect of pH and high pressure on N122-419-IB tertiary structure

Tryptophan (Trp) is an intrinsic fluorescence sensor for protein conformational changes, as the fluorescence peak shifts to higher wavelengths due to exposure of the hydrophobic residues to hydrophilic environment during denaturation.

SARS-CoV N protein presents five Trp and its fluorescence peak (λ_max_) was shown to be at 331–333 nm which suggests substantial burial of Trp residues from water. A shift to 354 nm was described to occur due to denaturation by incubation with 6M GdnHCl that promotes exposure of the Trp to the aqueous solvent [25,26]. Full-length SARS-CoV-2 N features five Trp (W52, W108, W132, W300 and W329) similarly to SARS-CoV N, while N122-419 lacks the first two Trp.

**Fig 3:**
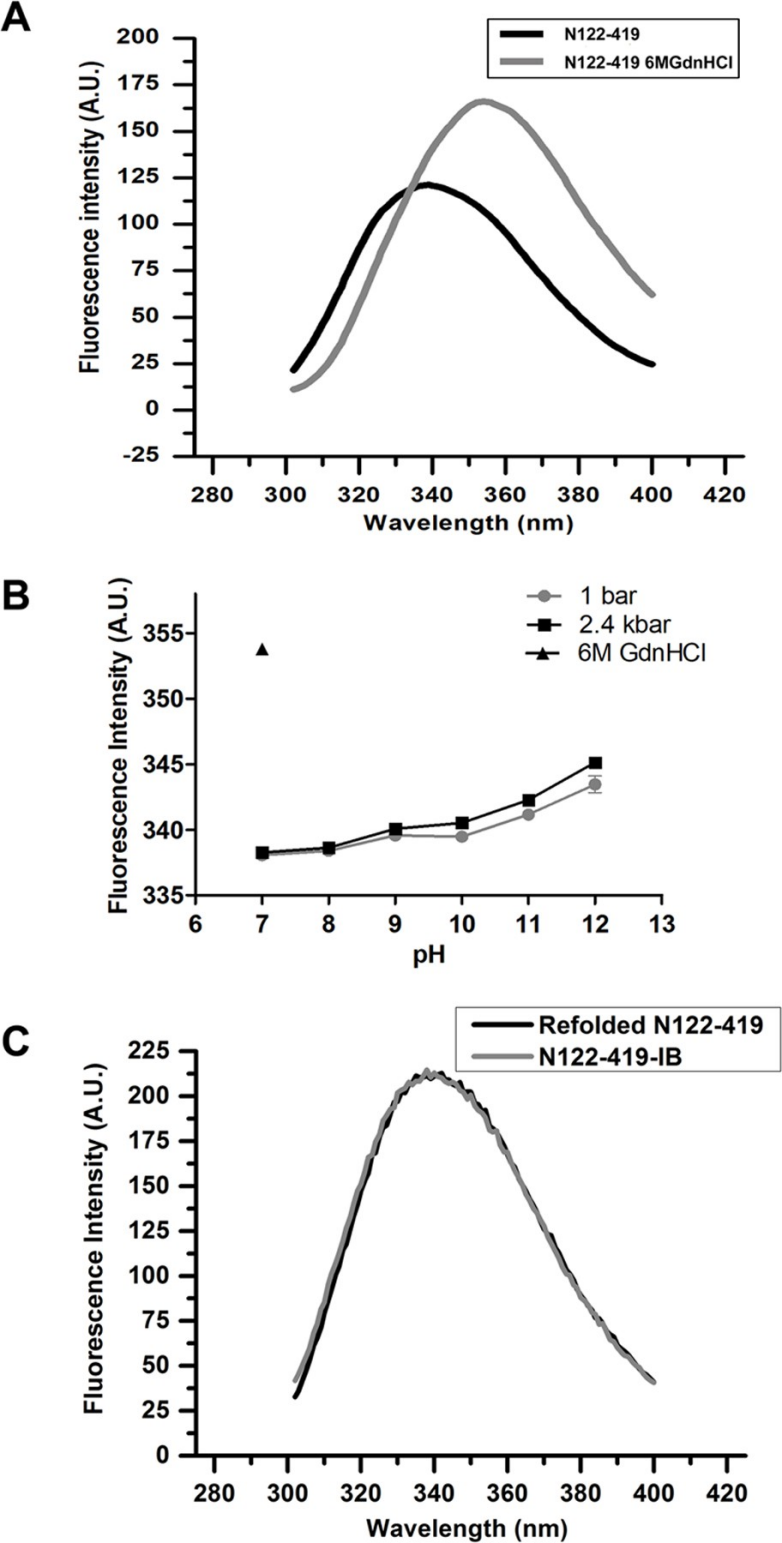
Compression at 2.4 kbar and alkaline pH induce only partial N122-419 unfolding. Suspensions of N122-419-IB were subjected to 2.4 kbar for 90 min or to 1 bar for 4 h. A, Spectra of fluorescence of N122-419-IB at pH 7.0 and in the presence of 6M GdnHCl; B, λ maximum vs pH. Each condition was analyzed in triplicate. Values are expressed as mean ± SD. The results are representative of 2 independent assays; C, Spectra of fluorescence of refolded N122-419 and of N122-419-IB. The intensity of fluorescence of N122-419-IB was normalized to reach a value similar to the spectrum of refolded protein, for a better comparison.

In order to monitor the unfolding process during solubilization, we measured the intrinsic fluorescence emission of N122-419 under various treatments. The suspension of N122-419-IB at pH 7.0 presents a fluorescence peak with a maximal value (λ_max_) at 338 nm. In the presence of 6M GdnHCl a 16 nm red shift in the λ_max_ to 354 nm was shown (Fig 3A), indicating that the protein in IB lost the tertiary structure by denaturation. The λ_max_ of N122-419 shifts to higher wavelengths as pH increase and the fluorescence of the protein subjected to 2.4 kbar showed a slightly higher degree of unfolding, up to 1 nm, when compared to samples maintained at 1 bar. Solubilization at pH 9 and 2.4 kbar induced a small λ_max_ shift of 2 nm to 340 nm (Fig 3B) in relation to the data obtained at pH 7.0 and 1 bar, indicating that this condition did not substantially unfold the protein.

N122-419 that was refolded by decreasing pH after solubilization at 2.4 kbar and pH 9.0, presents a λ_max_ at 338 nm (Fig 3C). This result suggests that the remaining Trp in N122-419 are more exposed to the solvent than in the full-length protein, therefore explaining the 6 nm shift of λ_max_ in comparison to the value described for SARS-CoV N (331–333 nm). Interestingly, the fluorescence profile and the λ_max_ of refolded N122-419 is very similar to the obtained for the suspension of N122-419-IB (Fig 3C) suggesting that, despite small changes of the intermolecular interactions that hold the architecture within N122-419-IB, the proteins in the IB present a native-like tertiary structure. In fact, we had observed in previous studies that the λ_max_ value of the protein expressed in IB is very similar to the native protein [19]. Therefore, it is likely that the solubilization using the extremely mild condition reduce exposure of hydrophobic residues of the protein, consequently decreasing undesirable intermolecular interactions and reaggregation.

### Immunological characterization of N122-419

**Fig 4:**
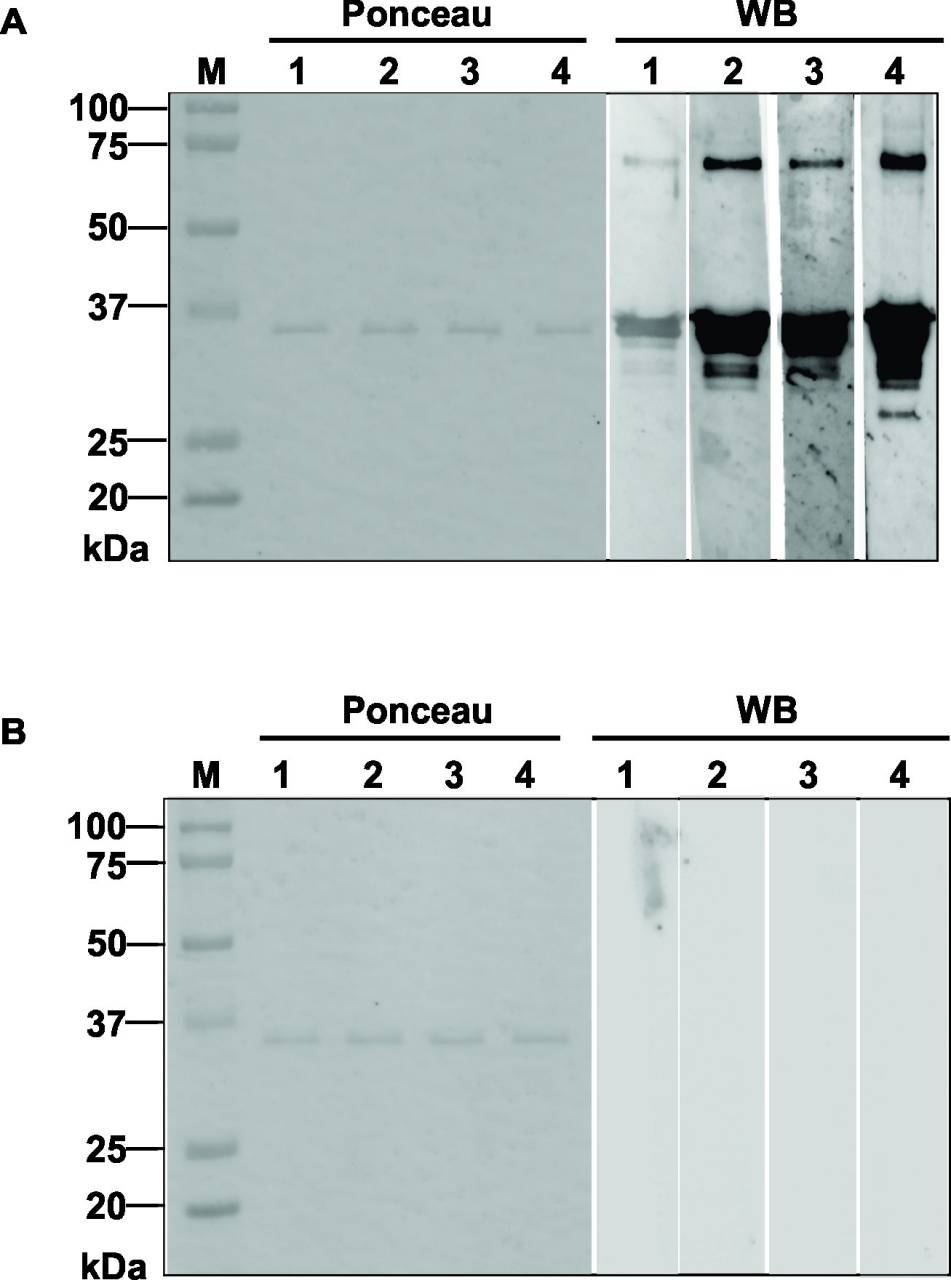
N122-419 is immunoreactive in Western blotting. N122-419 was subjected to SDS-PAGE, transferred to nitrocellulose membrane and to Western blotting using positive (A), and negative sera (B) for SARS-CoV-2.

N122-419 refolded at pH 9.0 and 2.4 kbar (non-purified) was shown to be immunoreactive at bands of 35 kDa and 70 kDa, which corresponds to N122-419 monomer and dimer respectively in Western blotting using positive sera for COVID-19 (Fig 4A). These bands were not observed when negative control sera were utilized (Fig 4B, columns 1–4).

### N122-419 is adequate for ELISA

**Fig 5:**
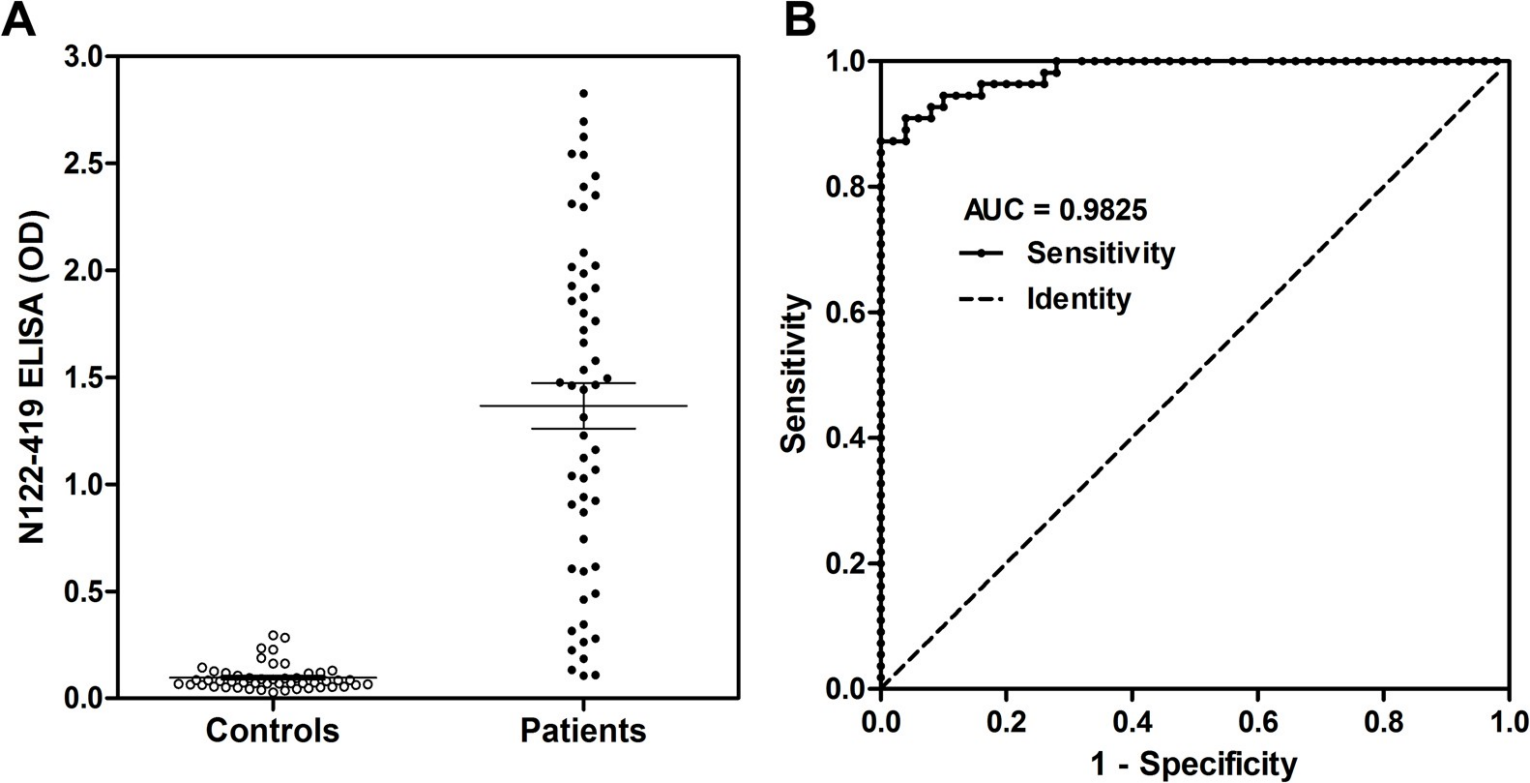
ELISA test is efficient for distinguishing between sera presenting antibodies against SARS-CoV-2 from those who do not. ROC curve results. A, Distribution of OD values from infected individuals and controls sample; B, Sensitivity and specificity rate for each cut-off point of the ROC curve and the AUC result.

The ELISA performance using the N122-149 protein as antigen was evaluated by the ROC curve (receiver operating characteristic) obtained by graphical representation of quantitative data based on the sensitivity rate (fraction of true positives) and the fraction of false positives (1-specificity). The OD values of samples from infected individuals and controls used to construct the ROC curve are shown in Fig 5A. The average OD of positive sera was significantly higher when compared to controls (p<0.0001). The Fig 5B shows the variation in sensitivity and specificity for each cut-off point of the ROC curve. In addition, the ELISA performance was also evaluated by the AUC (area under the curve) to estimate the overall accuracy of the test as it results from the integration of all points on the ROC curve, simultaneously computing sensitivity and specificity. The ELISA with the N122-149 protein showed a high AUC value (AUC = 0.9825; 95% CI: 0.9645–1.001) indicating that this diagnostic test is efficient for distinguishing between patients with antibodies against SARS-CoV-2 from people who do not [23].

## Discussion

*E*. *coli* bacteria are efficient hosts for the production of recombinant proteins. However, the advantage achieved by the simplicity of separating insoluble IB from soluble bacterial contaminants is often lost by low refolding yields due to protein reaggregation when removing the conditions utilized for IB solubilization. This problem occurs due to intermolecular interactions of hydrophobic patches that have been exposed during denaturing processes in IB solubilization. In the present study we describe a rapid and efficient non-denaturing method to obtain SARS-CoV-2 nucleocapsid. The refolded protein is suitable for use in immunodiagnostic assay to detect anti-SARS-CoV-2 antibodies.

Previous reports described obtainment of 3 and 30–55 mg recombinant SARS-CoV N/liter in *Escherichia coli* culture [27,28]. Contaminants are removed by metal affinity chromatography under denaturing conditions. To avoid aggregation, the concentration of the protein was reduced to below 100 μg/mL and it presents some degree of degradation as detected using SDS-PAGE [28].

Expression and refolding of SARS-CoV-2 N from IB produced in *E*. *coli* has also been described by Li et al [29]. Solubilization was performed under denaturing condition with 8M urea. However, two purification steps were necessary to obtain protein with 90% purity and the final yield was 50 mg N/liter of bacterial culture.

In the present study we obtained at least 6.5-fold higher yield (326 mg of refolded N122-419 with 95% purity from 1 L bacterial culture) than the previously described studies without the need for additional purification steps. The refolding process only takes 2 hours and pH adjustment is the only required manipulation after decompression. There is no requirement for dialysis because no additives are used during the process. Additionally, N122-419 presents no visible degradation when analyzed using SDS-PAGE.

**Fig 6:**
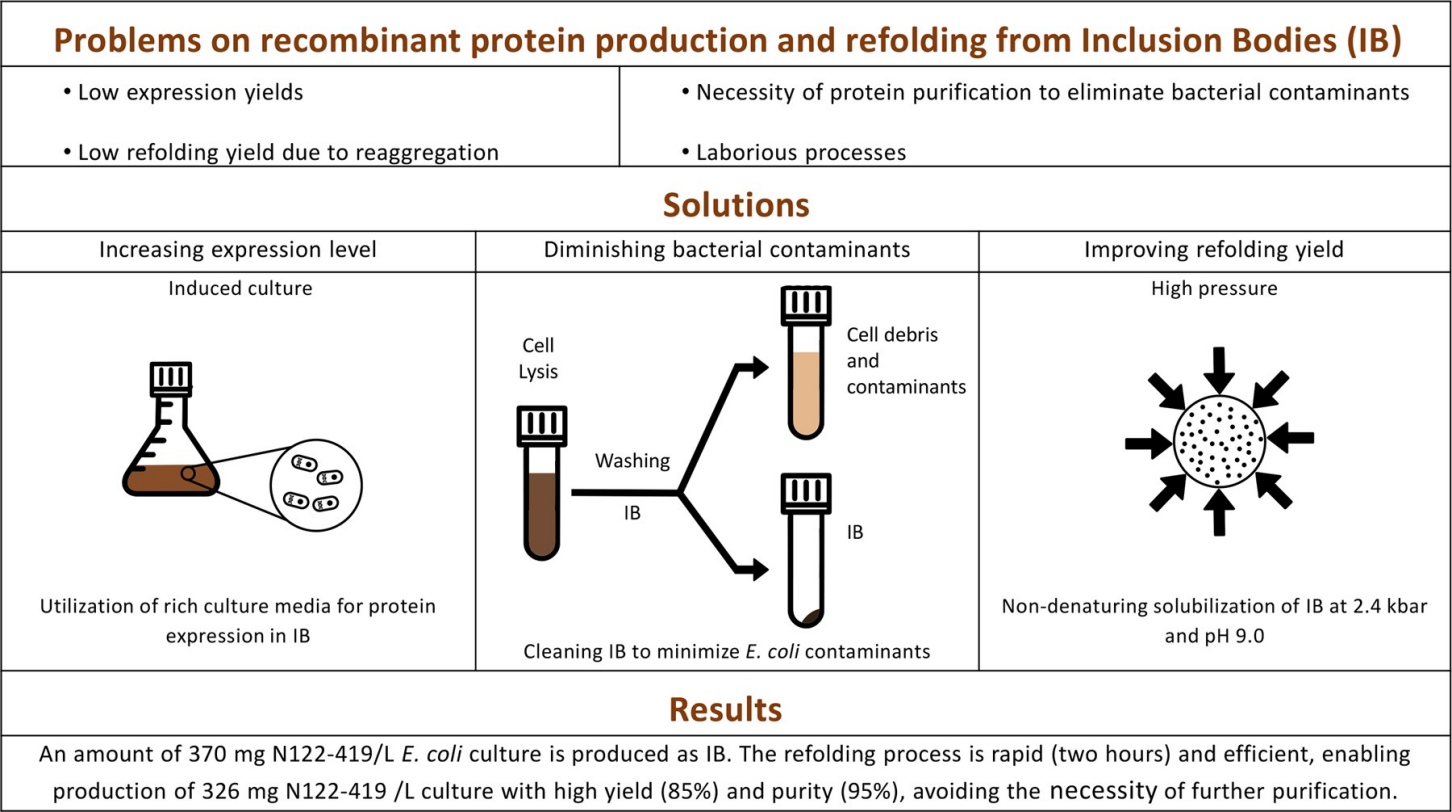
Scheme depicting the benefits of the utilization of the processes described in our study for expression of recombinant proteins, for washing inclusion bodies and for refolding.

We believed a number of factors contributed to our improved results (Fig 6):

- High protein expression. Besides the fact that the codons were optimized and that our expression system was efficient, the use of the rich medium for expression contributed to the high expression of N122-419 (369 mg/L culture) by the bacterial host.
- The washings of the IB were efficient, allowing high yields of N122-419-IB with a high degree of purity. This increased overall efficiency since additional downstream purification processes were not required.
- The use of enzyme inhibitor (PMSF) during IB washings and refolding and the rapid refolding process possibly inhibited proteolytic degradation since lower molecular weight products were not observed by SDS-PAGE in our study, unlike that described in the study of Das et al for SARS-CoV N [28].
- Finally, we believe that the most important factor for the achievement of a high refolding yield of N was the solubilization of IB in a non-denaturing condition. N122-419 in IB presents a native-like tertiary structure (Fig 3C) and mild solubilization, which maintains the hydrophobic regions of the protein unexposed, apparently avoids random intermolecular hydrophobic interactions. Thus, reaggregation, which negatively affects the refolding yield of proteins, was minimized.

Regarding the ELISA performance using the N122-419 protein as antigen, our data demonstrated that this test is efficient for detection of anti-SARS-CoV2 antibodies. These data corroborate the findings previously described by Meireles et al that demonstrated a greater sensitivity of ELISA with the N122-419 protein when compared to the test using total viral antigen [23].

## Conclusions

In conclusion, we developed a high-yield production method for N122-419 protein. This protein is suitable as antigen for in vitro tests in the determination of anti-SARS-CoV-2 antibodies. One liter of *E coli* culture yields sufficient amount for the preparation of 15,000 ELISA plates with 96 wells and 0.22 μg/well, or 600,000 duplicate tests.

The described process can possibly be extended to a wide range of proteins, achieving high yields by using an extremely fast refolding process that eliminates the need for purification.

## Supporting information

S1 Raw images (TIF)

S1 File Fig 2B data. (XLSX)

S2 File Fig 3A data. (XLSX)

S3 File Fig 3B data. (XLSX)

S4 File Fig 3C data. (XLSX)

S5 File Fig 5 data. (DOCX)

## Abbreviations

DTT: dithiothreitol
EDTA: ethylenediaminetetraacetic acid
ELISA: enzyme-linked immunosorbent assay
GdnHCl: guanidine hydrochloride
IPTG: isopropylβ-D-thiogalactopyranoside
LS: light scattering
N: Nucleocapsid
N122-419: SARS-CoV-2 fragment containing the residues 122 to 419
N122-419-IB: N122-419-Inclusion Bodies
PMSF: Phenylmethanesulfonyl fluoride
SDS-PAGE: sodium dodecyl sulphate polyacrylamide gel electrophoresis
