# Correction: High level SARS-CoV-2 nucleocapsid refolding using mild condition for inclusion bodies solubilization: Application of high pressure at pH 9.0

**DOI:** 10.1371/journal.pone.0271140

**Published:** 2022-07-05

**Authors:** The PLOS ONE Staff

The Funding statement is incorrect. The correct Funding statement is as follows: This study was supported by Fundação de Amparo à Pesquisa do Estado de São Paulo (FAPESP) grant 2015/02574-0 (LM). This study was also supported by Coordenação de Aperfeiçoamento de Pessoal de Nível Superior (CAPES) postdoctoral fellowship grant 88887.334462/2019-00 (RMC-C). The funders had no role in study design, data collection and analysis, decision to publish, or preparation of the manuscript. The publisher apologizes for the error.
