# Supplementary figures and images for: High level SARS-CoV-2 nucleocapsid refolding using mild condition for inclusion bodies solubilization: Application of high pressure at pH 9.0
Source: PLoS One. 2022 Feb 3;17(2):e0262591. doi: 10.1371/journal.pone.0262591 (PMC8812862; doi:10.1371/journal.pone.0262591)

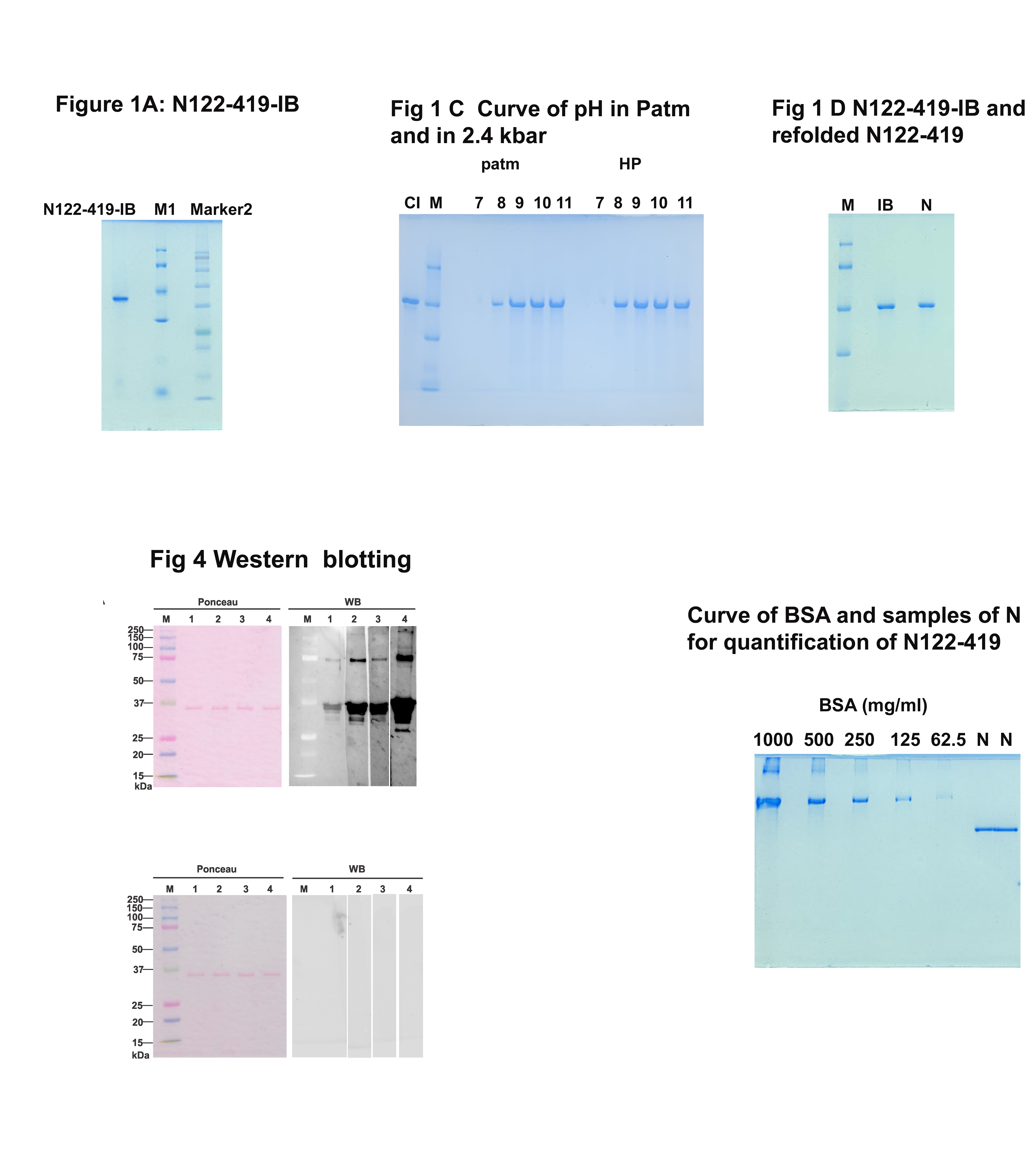

Supplement: S1 Raw images — (TIF) [file pone.0262591.s001.tif]
