# Supplementary material for: High level SARS-CoV-2 nucleocapsid refolding using mild condition for inclusion bodies solubilization: Application of high pressure at pH 9.0
Source: PLoS One. 2022 Feb 3;17(2):e0262591. doi: 10.1371/journal.pone.0262591 (PMC8812862; doi:10.1371/journal.pone.0262591)
Supplement: S5 File — (DOCX) [file pone.0262591.s006.docx]

1. **Data of ROC curve (OD)**

| Controls | Patients |
| --- | --- |
| 0,051500 | 1,578000 |
| 0,065500 | 2,828000 |
| 0,143000 | 0,185000 |
| 0,088500 | 0,278500 |
| 0,071000 | 0,593000 |
| 0,106000 | 0,606500 |
| 0,163000 | 2,295000 |
| 0,283000 | 1,314500 |
| 0,063500 | 1,985500 |
| 0,043500 | 1,029000 |
| 0,085500 | 0,743500 |
| 0,069000 | 0,105500 |
| 0,094000 | 1,161500 |
| 0,233500 | 1,040000 |
| 0,084500 | 1,465500 |
| 0,077000 | 0,314500 |
| 0,036500 | 0,263500 |
| 0,038500 | 0,906000 |
| 0,079500 | 2,016500 |
| 0,227500 | 0,108000 |
| 0,083500 | 2,083500 |
| 0,080000 | 0,461000 |
| 0,163500 | 1,720000 |
| 0,119000 | 0,132000 |
| 0,054000 | 2,311500 |
| 0,188000 | 1,228500 |
| 0,050000 | 1,877000 |
| 0,094000 | 1,535000 |
| 0,117500 | 0,615000 |
| 0,294000 | 1,123000 |
| 0,090000 | 1,476000 |
| 0,120000 | 2,390500 |
| 0,129500 | 0,923000 |
| 0,067500 | 0,941500 |
| 0,126000 | 0,224500 |
| 0,065000 | 1,443500 |
| 0,074500 | 1,917500 |
| 0,055000 | 1,799500 |
| 0,074500 | 1,462000 |
| 0,069500 | 0,346000 |
| 0,093000 | 0,870000 |
| 0,062000 | 2,022500 |
| 0,070000 | 1,763000 |
| 0,070000 | 1,495000 |
| 0,052500 | 0,489500 |
| 0,052000 | 1,067500 |
| 0,044000 | 2,625000 |
| 0,047000 | 2,352000 |
| 0,027500 | 2,545000 |
| 0,084000 | 1,858000 |
|  | 1,662000 |
|  | 2,696000 |
|  | 2,442000 |
|  | 1,928000 |
|  | 2,539500 |

1. **Determination of Area under the ROC curve**

| Area under the ROC curve | Results |
| --- | --- |
| Area | 0,9825 |
| Std. Error | 0,009181 |
| 95% confidence interval | 0.9645 to 1.001 |
| P value | < 0.0001 |
| Data |  |
| Control | 50 |
| Patient | 55 |
| Missing Controls | 0 |
| Missing Patients | 0 |
|  |  |

1. **Data for determination of sensitivity, specificity and likelihood ratio**

|  | Sensitivity | 95% CI | Specificity | 95% CI | Likelihood ratio |
| --- | --- | --- | --- | --- | --- |
| > 0.0320 | 1,000 | 0.9351 to 1.000 | 0,0200 | 0.0005062 to 0.1065 | 1,02 |
| > 0.0375 | 1,000 | 0.9351 to 1.000 | 0,0400 | 0.004881 to 0.1371 | 1,04 |
| > 0.0410 | 1,000 | 0.9351 to 1.000 | 0,0600 | 0.01255 to 0.1655 | 1,06 |
| > 0.04375 | 1,000 | 0.9351 to 1.000 | 0,0800 | 0.02223 to 0.1923 | 1,09 |
| > 0.0455 | 1,000 | 0.9351 to 1.000 | 0,1000 | 0.03328 to 0.2181 | 1,11 |
| > 0.0485 | 1,000 | 0.9351 to 1.000 | 0,1200 | 0.04534 to 0.2431 | 1,14 |
| > 0.05075 | 1,000 | 0.9351 to 1.000 | 0,1400 | 0.05819 to 0.2674 | 1,16 |
| > 0.05175 | 1,000 | 0.9351 to 1.000 | 0,1600 | 0.07170 to 0.2911 | 1,19 |
| > 0.05225 | 1,000 | 0.9351 to 1.000 | 0,1800 | 0.08576 to 0.3144 | 1,22 |
| > 0.05325 | 1,000 | 0.9351 to 1.000 | 0,2000 | 0.1003 to 0.3372 | 1,25 |
| > 0.0545 | 1,000 | 0.9351 to 1.000 | 0,2200 | 0.1153 to 0.3596 | 1,28 |
| > 0.0585 | 1,000 | 0.9351 to 1.000 | 0,2400 | 0.1306 to 0.3817 | 1,32 |
| > 0.06275 | 1,000 | 0.9351 to 1.000 | 0,2600 | 0.1463 to 0.4035 | 1,35 |
| > 0.06425 | 1,000 | 0.9351 to 1.000 | 0,2800 | 0.1623 to 0.4249 | 1,39 |
| > 0.06525 | 1,000 | 0.9351 to 1.000 | 0,3000 | 0.1786 to 0.4461 | 1,43 |
| > 0.0665 | 1,000 | 0.9351 to 1.000 | 0,3200 | 0.1952 to 0.4670 | 1,47 |
| > 0.06825 | 1,000 | 0.9351 to 1.000 | 0,3400 | 0.2121 to 0.4877 | 1,52 |
| > 0.06925 | 1,000 | 0.9351 to 1.000 | 0,3600 | 0.2292 to 0.5081 | 1,56 |
| > 0.06975 | 1,000 | 0.9351 to 1.000 | 0,3800 | 0.2465 to 0.5282 | 1,61 |
| > 0.0705 | 1,000 | 0.9351 to 1.000 | 0,4200 | 0.2819 to 0.5679 | 1,72 |
| > 0.07275 | 1,000 | 0.9351 to 1.000 | 0,4400 | 0.2999 to 0.5875 | 1,79 |
| > 0.07575 | 1,000 | 0.9351 to 1.000 | 0,4800 | 0.3366 to 0.6258 | 1,92 |
| > 0.07825 | 1,000 | 0.9351 to 1.000 | 0,5000 | 0.3553 to 0.6447 | 2,00 |
| > 0.07975 | 1,000 | 0.9351 to 1.000 | 0,5200 | 0.3742 to 0.6634 | 2,08 |
| > 0.08175 | 1,000 | 0.9351 to 1.000 | 0,5400 | 0.3932 to 0.6819 | 2,17 |
| > 0.08375 | 1,000 | 0.9351 to 1.000 | 0,5600 | 0.4125 to 0.7001 | 2,27 |
| > 0.08425 | 1,000 | 0.9351 to 1.000 | 0,5800 | 0.4321 to 0.7181 | 2,38 |
| > 0.0850 | 1,000 | 0.9351 to 1.000 | 0,6000 | 0.4518 to 0.7359 | 2,50 |
| > 0.0870 | 1,000 | 0.9351 to 1.000 | 0,6200 | 0.4718 to 0.7535 | 2,63 |
| > 0.08925 | 1,000 | 0.9351 to 1.000 | 0,6400 | 0.4919 to 0.7708 | 2,78 |
| > 0.0915 | 1,000 | 0.9351 to 1.000 | 0,6600 | 0.5123 to 0.7879 | 2,94 |
| > 0.0935 | 1,000 | 0.9351 to 1.000 | 0,6800 | 0.5330 to 0.8048 | 3,13 |
| > 0.09975 | 1,000 | 0.9351 to 1.000 | 0,7200 | 0.5751 to 0.8377 | 3,57 |
| > 0.1058 | 0,9818 | 0.9028 to 0.9995 | 0,7200 | 0.5751 to 0.8377 | 3,51 |
| > 0.1070 | 0,9818 | 0.9028 to 0.9995 | 0,7400 | 0.5965 to 0.8537 | 3,78 |
| > 0.1128 | 0,9636 | 0.8747 to 0.9956 | 0,7400 | 0.5965 to 0.8537 | 3,71 |
| > 0.1183 | 0,9636 | 0.8747 to 0.9956 | 0,7600 | 0.6183 to 0.8694 | 4,02 |
| > 0.1195 | 0,9636 | 0.8747 to 0.9956 | 0,7800 | 0.6404 to 0.8847 | 4,38 |
| > 0.1230 | 0,9636 | 0.8747 to 0.9956 | 0,8000 | 0.6628 to 0.8997 | 4,82 |
| > 0.1278 | 0,9636 | 0.8747 to 0.9956 | 0,8200 | 0.6856 to 0.9142 | 5,35 |
| > 0.1308 | 0,9636 | 0.8747 to 0.9956 | 0,8400 | 0.7089 to 0.9283 | 6,02 |
| > 0.1375 | 0,9455 | 0.8488 to 0.9886 | 0,8400 | 0.7089 to 0.9283 | 5,91 |
| > 0.1530 | 0,9455 | 0.8488 to 0.9886 | 0,8600 | 0.7326 to 0.9418 | 6,75 |
| > 0.1633 | 0,9455 | 0.8488 to 0.9886 | 0,8800 | 0.7569 to 0.9547 | 7,88 |
| > 0.1743 | 0,9455 | 0.8488 to 0.9886 | 0,9000 | 0.7819 to 0.9667 | 9,45 |
| > 0.1865 | 0,9273 | 0.8241 to 0.9798 | 0,9000 | 0.7819 to 0.9667 | 9,27 |
| > 0.2063 | 0,9273 | 0.8241 to 0.9798 | 0,9200 | 0.8077 to 0.9778 | 11,59 |
| > 0.2260 | 0,9091 | 0.8005 to 0.9698 | 0,9200 | 0.8077 to 0.9778 | 11,36 |
| > 0.2305 | 0,9091 | 0.8005 to 0.9698 | 0,9400 | 0.8345 to 0.9875 | 15,15 |
| > 0.2485 | 0,9091 | 0.8005 to 0.9698 | 0,9600 | 0.8629 to 0.9951 | 22,73 |
| > 0.2710 | 0,8909 | 0.7775 to 0.9589 | 0,9600 | 0.8629 to 0.9951 | 22,27 |
| > 0.2808 | 0,8727 | 0.7552 to 0.9473 | 0,9600 | 0.8629 to 0.9951 | 21,82 |
| > 0.2885 | 0,8727 | 0.7552 to 0.9473 | 0,9800 | 0.8935 to 0.9995 | 43,64 |
| > 0.3043 | 0,8727 | 0.7552 to 0.9473 | 1,000 | 0.9289 to 1.000 |  |
| > 0.3303 | 0,8545 | 0.7334 to 0.9350 | 1,000 | 0.9289 to 1.000 |  |
| > 0.4035 | 0,8364 | 0.7120 to 0.9223 | 1,000 | 0.9289 to 1.000 |  |
| > 0.4753 | 0,8182 | 0.6909 to 0.9092 | 1,000 | 0.9289 to 1.000 |  |
| > 0.5413 | 0,8000 | 0.6703 to 0.8957 | 1,000 | 0.9289 to 1.000 |  |
| > 0.5998 | 0,7818 | 0.6499 to 0.8819 | 1,000 | 0.9289 to 1.000 |  |
| > 0.6108 | 0,7636 | 0.6298 to 0.8677 | 1,000 | 0.9289 to 1.000 |  |
| > 0.6793 | 0,7455 | 0.6100 to 0.8533 | 1,000 | 0.9289 to 1.000 |  |
| > 0.8068 | 0,7273 | 0.5904 to 0.8386 | 1,000 | 0.9289 to 1.000 |  |
| > 0.8880 | 0,7091 | 0.5710 to 0.8237 | 1,000 | 0.9289 to 1.000 |  |
| > 0.9145 | 0,6909 | 0.5519 to 0.8086 | 1,000 | 0.9289 to 1.000 |  |
| > 0.9323 | 0,6727 | 0.5329 to 0.7932 | 1,000 | 0.9289 to 1.000 |  |
| > 0.9853 | 0,6545 | 0.5142 to 0.7776 | 1,000 | 0.9289 to 1.000 |  |
| > 1.035 | 0,6364 | 0.4956 to 0.7619 | 1,000 | 0.9289 to 1.000 |  |
| > 1.054 | 0,6182 | 0.4773 to 0.7459 | 1,000 | 0.9289 to 1.000 |  |
| > 1.095 | 0,6000 | 0.4591 to 0.7298 | 1,000 | 0.9289 to 1.000 |  |
| > 1.142 | 0,5818 | 0.4411 to 0.7135 | 1,000 | 0.9289 to 1.000 |  |
| > 1.195 | 0,5636 | 0.4232 to 0.6970 | 1,000 | 0.9289 to 1.000 |  |
| > 1.272 | 0,5455 | 0.4055 to 0.6803 | 1,000 | 0.9289 to 1.000 |  |
| > 1.379 | 0,5273 | 0.3880 to 0.6635 | 1,000 | 0.9289 to 1.000 |  |
| > 1.453 | 0,5091 | 0.3707 to 0.6465 | 1,000 | 0.9289 to 1.000 |  |
| > 1.464 | 0,4909 | 0.3535 to 0.6293 | 1,000 | 0.9289 to 1.000 |  |
| > 1.471 | 0,4727 | 0.3365 to 0.6120 | 1,000 | 0.9289 to 1.000 |  |
| > 1.486 | 0,4545 | 0.3197 to 0.5945 | 1,000 | 0.9289 to 1.000 |  |
| > 1.515 | 0,4364 | 0.3030 to 0.5768 | 1,000 | 0.9289 to 1.000 |  |
| > 1.557 | 0,4182 | 0.2865 to 0.5589 | 1,000 | 0.9289 to 1.000 |  |
| > 1.620 | 0,4000 | 0.2702 to 0.5409 | 1,000 | 0.9289 to 1.000 |  |
| > 1.691 | 0,3818 | 0.2541 to 0.5227 | 1,000 | 0.9289 to 1.000 |  |
| > 1.742 | 0,3636 | 0.2381 to 0.5044 | 1,000 | 0.9289 to 1.000 |  |
| > 1.781 | 0,3455 | 0.2224 to 0.4858 | 1,000 | 0.9289 to 1.000 |  |
| > 1.829 | 0,3273 | 0.2068 to 0.4671 | 1,000 | 0.9289 to 1.000 |  |
| > 1.868 | 0,3091 | 0.1914 to 0.4481 | 1,000 | 0.9289 to 1.000 |  |
| > 1.897 | 0,2909 | 0.1763 to 0.4290 | 1,000 | 0.9289 to 1.000 |  |
| > 1.923 | 0,2727 | 0.1614 to 0.4096 | 1,000 | 0.9289 to 1.000 |  |
| > 1.957 | 0,2545 | 0.1467 to 0.3900 | 1,000 | 0.9289 to 1.000 |  |
| > 2.001 | 0,2364 | 0.1323 to 0.3702 | 1,000 | 0.9289 to 1.000 |  |
| > 2.020 | 0,2182 | 0.1181 to 0.3501 | 1,000 | 0.9289 to 1.000 |  |
| > 2.053 | 0,2000 | 0.1043 to 0.3297 | 1,000 | 0.9289 to 1.000 |  |
| > 2.189 | 0,1818 | 0.09079 to 0.3091 | 1,000 | 0.9289 to 1.000 |  |
| > 2.303 | 0,1636 | 0.07766 to 0.2880 | 1,000 | 0.9289 to 1.000 |  |
| > 2.332 | 0,1455 | 0.06495 to 0.2666 | 1,000 | 0.9289 to 1.000 |  |
| > 2.371 | 0,1273 | 0.05274 to 0.2448 | 1,000 | 0.9289 to 1.000 |  |
| > 2.416 | 0,1091 | 0.04110 to 0.2225 | 1,000 | 0.9289 to 1.000 |  |
| > 2.491 | 0,09091 | 0.03018 to 0.1995 | 1,000 | 0.9289 to 1.000 |  |
| > 2.542 | 0,07273 | 0.02017 to 0.1759 | 1,000 | 0.9289 to 1.000 |  |
| > 2.585 | 0,05455 | 0.01139 to 0.1512 | 1,000 | 0.9289 to 1.000 |  |
| > 2.661 | 0,03636 | 0.004435 to 0.1253 | 1,000 | 0.9289 to 1.000 |  |
| > 2.762 | 0,01818 | 0.0004602 to 0.09719 | 1,000 | 0.9289 to 1.000 |  |
